# Heterologous Prime-Boost HIV-1 Vaccination Regimens in Pre-Clinical and Clinical Trials

**DOI:** 10.3390/v2020435

**Published:** 2010-02-01

**Authors:** Scott A. Brown, Sherri L. Surman, Robert Sealy, Bart G. Jones, Karen S. Slobod, Kristen Branum, Timothy D. Lockey, Nanna Howlett, Pamela Freiden, Patricia Flynn, Julia L. Hurwitz

**Affiliations:** 1 Department of Immunology, St. Jude Children’s Research Hospital, 262 Danny Thomas Place, Memphis, TN, USA; E-Mail: scott.brown@stjude.org (S.A.B.); 2 Department of Infectious Diseases, St. Jude Children’s Research Hospital, 262 Danny Thomas Place, Memphis, TN, USA; E-Mails: sherri.surman@stjude.org (S.L.S.); bob.sealy@stjude.org (R.S.); bart.jones@stjude.org (B.G.J.); kristen.branum@stjude.org (K.B.); nanna.howlett@stjude.org (N.H.); pamela.freiden@stjude.org (P.F.); patricia.flynn@stjude.org (P.F.); 3 Early Development, Novartis Vaccines and Diagnostics, 350 Mass Ave. Cambridge, MA 02139, USA; E-Mail: karen.slobod@novartis.com (K.S.S.); 4 Department of Therapeutics, Production and Quality, St. Jude Children’s Research Hospital, 262 Danny Thomas Place, Memphis, TN, USA; E-Mail: timothy.lockey@stjude.org (T.D.L.); 5 Department of Pediatrics, University of Tennessee, Memphis, TN 38163, USA; 6 Department of Pathology, University of Tennessee, Memphis, TN 38163, USA

**Keywords:** HIV-1, prime-boost, heterologous, Sendai virus, clinical trials

## Abstract

Currently, there are more than 30 million people infected with HIV-1 and thousands more are infected each day. Vaccination is the single most effective mechanism for prevention of viral disease, and after more than 25 years of research, one vaccine has shown somewhat encouraging results in an advanced clinical efficacy trial. A modified intent-to-treat analysis of trial results showed that infection was approximately 30% lower in the vaccine group compared to the placebo group. The vaccine was administered using a heterologous prime-boost regimen in which both target antigens and delivery vehicles were changed during the course of inoculations. Here we examine the complexity of heterologous prime-boost immunizations. We show that the use of different delivery vehicles in prime and boost inoculations can help to avert the inhibitory effects caused by vector-specific immune responses. We also show that the introduction of new antigens into boost inoculations can be advantageous, demonstrating that the effect of ‘original antigenic sin’ is not absolute. Pre-clinical and clinical studies are reviewed, including our own work with a three-vector vaccination regimen using recombinant DNA, virus (Sendai virus or vaccinia virus) and protein. Promising preliminary results suggest that the heterologous prime-boost strategy may possibly provide a foundation for the future prevention of HIV-1 infections in humans.

## 1. Introduction

Tens of millions of individuals are currently living with HIV-1. In the United States alone, despite plentiful resources, there are an estimated one million individuals infected with HIV-1, and approximately 55,000 new infections each year [1,2]. Vaccination remains among the highest health priorities, and despite many past disappointments in HIV-1 vaccine clinical trials, recent results from a phase III study suggest that progress is being made toward the development of a protective product [3–7]. The trial was named RV 144 and was conducted in Thailand with more than 16,000 study participants. The vaccine regimen employed a heterologous prime-boost strategy. One vaccine component was a recombinant canarypox vector, ALVAC-HIV (vCP1521), produced by Aventis Pasteur. This vector expressed HIV-1 envelope (from CRF01_AE 92TH023 and LAI viruses), gag (LAI) and protease (LAI) sequences. The second vaccine component was a mixture of two CHO-derived HIV-1 envelope proteins formulated in alum, named AIDSVAX B/E, produced by Vaxgen. These protein sequences derived from CRF01_AE A244 and MN [8]. Trial participants were first inoculated with vCP1521 on weeks 0 and 4. They were then inoculated with vCP1521 mixed with AIDSVAX B/E on weeks 12 and 24. When the outcome of vaccinations was evaluated by a modified intent-to-treat analysis, results showed that there were approximately 30% fewer infections in the vaccinated group compared to the placebo control group. These somewhat encouraging results provide a stepping stone for the production of improved vaccines. Here we will examine the obstacles presented by HIV-1 and the means by which a heterologous prime-boost vaccine might overcome them.

## 2. The obstacles presented by HIV-1

### 2.1. Antigen diversity

One of the biggest challenges in HIV-1 vaccine design is posed by the striking diversity of HIV-1 proteins. This virus mutates at an impressive rate due to its error-prone reverse transcriptase and lack of proof-reading capacity [9–11]. The envelope protein is most diverse [10–12], but none of the viral proteins, either internal or external, are entirely conserved. Animal studies have shown repeatedly that HIV-1 readily escapes a focused immune response, whether B-cell or T-cell mediated [13,14].

One might be inclined to describe the diversity of HIV-1 proteins as limitless, but requirements for protein function impose some limits. For example, the envelope protein must bind the highly conserved CD4 and co-receptor molecules to mediate the fusion of virus and host cell target membranes [15]. These requirements levy constraints on the number of mutually exclusive three-dimensional structures that a functional protein can assume.

#### 2.1.1. Conserved determinants within HIV-1 as targets for vaccine design

A popular strategy in the HIV-1 field is to identify a B-cell or T-cell determinant that is conserved or semi-conserved across all HIV-1 isolates and to mark this as a central target for vaccine design. If a conserved determinant can be associated with a protective response and then rendered immunogenic in all humans, this could be the basis of a successful vaccine. In the 1990s, for example, vaccine developers began to target semi-conserved determinants in the V3 loop [16,17], once termed the principal neutralizing determinant (PND) of HIV-1. Numerous additional neutralizing determinants have since been targeted [18–21]. Once a conserved determinant is recognized, researchers seek methods to present that determinant to the immune system, in some cases by unmasking the determinant via HIV-1 gene manipulation (e.g., by removal of a variable region on the envelope protein [22,23] or by stabilization of an envelope-CD4 fusion intermediate [24,25]).

Attempts to render a conserved determinant immunogenic for all humans have not yet been successful, a situation which may reflect the many years of co-evolution between HIV-1 and its human host. In essence, evolution has selected HIV-1 proteins that accept variation among important B-cell and T-cell target epitopes and can thus escape a focused immune response. Conserved determinants, by definition, are those that resist immune pressure. Either their recognition by the immune system is inconsequential to virus growth, or they evade immune responses (e.g., neutralizing antibodies) by (i) concealment beneath variable protein fragments or sugar groups, or (ii) mimicry of ‘self’ proteins toward which humans are tolerant [26]. The unfortunate outcome is that protective immune responses toward conserved HIV-1 determinants are rare in healthy humans and difficult to induce.

#### 2.1.2. Harnessing diverse lymphocytes to target a diverse pathogen

An alternative strategy, based on impressive successes in other vaccine fields, is to formulate vaccine cocktails representative of pathogen diversity. This strategy is aimed at harnessing the full capacity of the immune system rather than a few rare antibodies or T-cell receptors (TCR). Indeed, the strength of the immune system lies in its capacity to respond to an enormous repertoire of antigens. A sophisticated mechanism of rearrangement among immunoglobulin or TCR variable, diversity, joining and constant region genes makes this possible, as each combination yields a different receptor with capacity to recognize a different pathogen or foreign particle via a lock-and-key type interaction. The number of distinct receptors is further increased by imprecise gene joining mechanisms as well as n-and p-region additions [27]. Because of the impressive diversity of receptors in the immune system, virtually every pathogen in nature can be countered by a ‘specific’ set of B-cell and T-cell populations. It may be particularly useful to harness this army of immune effectors when tackling a diverse pathogen like HIV-1.

To create vaccine cocktails, researchers in other fields generally group pathogens by antigenic structure, most often defined by antibody recognition patterns. Once antigens are grouped, representative members from each group are assembled into vaccines to activate respective B-cell and T-cell populations. It is noteworthy that in past years, antigenicity was generally defined by functional assays rather than by evaluation of amino acid similarities/differences between two proteins. This was in part due to technological limitations, but provided some advantage given that (i) two proteins may differ at only one amino acid, but may assume different 3-dimensional antigenic structures, and (ii) conversely, two proteins with multiple amino acid changes may be structurally and antigenically alike [28].

The individual antibodies induced by cocktail vaccines need not be ‘broadly-neutralizing’ provided that they function together to target the breadth of pathogen diversity [29]. As examples, cocktail approaches have been successfully employed to tackle polio virus (trivalent), seasonal influenza virus (trivalent), rotavirus (pentavalent [30]), papillomavirus (quadravalent[31]) and pneumococcus (7,9,10,11 or 23-valent [32,33]). In the HIV-1 vaccine field, cocktail approaches are gaining favor. The vaccine in the recent RV 144 clinical trial included proteins from several different viruses and conferred a hint of protection to vaccinees. Perhaps larger cocktail vaccines, currently under development, will enhance protective efficacy in future clinical trials.

### 2.2. The chronic nature of HIV-1 infection

The chronic nature of HIV-1 infection is another obstacle often perceived as a major hindrance to HIV-1 vaccine development. However, this feature is perhaps more problematic for the development of viral therapies than for the development of vaccines. In recent years, successful vaccines have been developed and licensed against viruses that are associated with chronic infection, even though the development of therapies or cures has been difficult (e.g., Varicella zoster virus and papilloma virus [31,34]). Like HIV-1, these viruses can persist for long periods without disease, co-existing with the immune response. However, an insult to the immune system (whether related or unrelated to the virus infection) can associate with virus flare, sometimes with a fatal outcome [35,36].

The complexity of chronic virus disease highlights the importance of preventive measures, as it is far easier to block virus at the moment of first exposure than after the establishment of chronic infection. The history of effective vaccines against herpes and papilloma viruses demonstrates that even when certain viruses cannot be cleared from infected humans, successful vaccines can be designed.

## 3. HIV-1 vaccine development: non recombinant vaccine strategies

### 3.1. Killed vaccines

One of the first vaccines to be tested in the HIV-1 field was the killed virus vaccine. At first glance, this strategy appeared successful when tested in an SIV macaque model [37], but excitement turned to disappointment when it was discovered that the protective response was a consequence of experimental design. As it turned out, the vaccine and challenge virus stocks had been propagated on human cells and the protective immune responses in macaques were generated toward human MHC proteins that were incorporated into viral membranes [38,39]. An additional disappointment came when certain viral purification methods were found to release envelope proteins from the virus surface. Finally, there was the lingering concern that virus inactivation might not be 100% complete and that ‘killed’ HIV-1 vaccines might infect healthy volunteers. Despite the original disappointments associated with the killed vaccine approach, research continues in this area. Inactivation and purification procedures have been refined so that there is convincing kill with retention of immunogenic envelope proteins [40]. Perhaps these results will one day renew enthusiasm for advanced testing of the killed HIV-1 vaccine approach.

### 3.2. Attenuated Virus Vaccines

The attenuation of virus was a second method used in early research in the HIV-1 field. As with the killed vaccine approach, first results were promising. A nef-deletion mutant was produced that protected macaques from SIV infection, provided that there was a several month delay between vaccination and challenge [41]. However, success was again transient. Disappointment came when a fraction of animals infected with the attenuated virus vaccines experienced morbidity and mortality [42]. These incidents validated previous concerns that attenuated vaccines might not be safe [43,44]. The virus was capable of repairing the nef deletion mutation and reverting to wildtype phenotype [43,45]. Investigators next sought to strike a balance between virus attenuation and immunogenicity by introducing additional mutations into vaccines. Enhanced safety was achieved when double or triple mutations were introduced into the virus genome, but this additional crippling of virus replication also resulted in weaker immune responses that failed to confer complete protective immunity [46].

Numerous laboratories have now demonstrated that infection with one immunodeficiency virus (SIV or SHIV) can protect against subsequent infection with another [47–50]. How can this be explained? Perhaps the protection induced by live virus vaccines is dependent on a complex interplay between virus and the immune response. Once a virus establishes itself in a safe sanctuary (e.g. in the brain) it may mutate repeatedly. Over the course of months, the immune system is therefore exposed to a cocktail of new antigens created by escape mutation [14]. Possibly, cocktails induce a variety of heterogeneous B cells and T cells, each with a different antigenic specificity and each able to tackle a different virus subset. Together the lymphocytes work as an army [29] to survey the full population of variant viruses. Viral infections from an exogenous source may then be blocked, because each challenge virus matches at least one of the escape variants in the natural vaccine by antigenicity.

This explanation for virus-induced protective immunity might explain the failure of the double- or triple-deletion viral vaccines. When virus growth is severely attenuated by the introduction of multiple mutations, the generation of escape mutants is curtailed and diverse antigens do not evolve. The vaccine is no longer a cocktail, and responding lymphocytes are not diverse. Vaccine efficacy is lost when a focused response cannot recognize the numerous viral variants of nature.

As with the killed vaccine, the attenuated virus vaccine approach remains a topic of active research. Scientists will continue to seek a balance between vaccine safety and the induction of robust, heterogeneous immune cells.

## 4. Recombinant vaccine strategies

### 4.1. Introduction of recombinant vectors

The discovery of HIV-1 as the cause of AIDS coincided temporally with an explosive use of recombinant nucleic acid technology [51–53]. It is not surprising that vaccine developers harnessed this technology to create recombinant vaccines with dozens of different vector systems. To date, recombinant SIV or HIV-1 vaccine products have been based on vectors including leishmania [54], baculovirus [55], Semlicki Forest virus [56,57], Venezuelan equine encephalitis virus [58], adenovirus [59], adeno-associated virus [60,61], vaccinia virus [62], modified vaccinia ankara (MVA [63,64]), canarypox, fowlpox [65–67], yeast [68], vesicular stomatitis virus [69], *Listeria monocytogenes* [70], phage [71], ovine atadenovirus [72], *Mycobacterium tuberculosis* [73], foamy virus [74], influenza virus [75], coxsackievirus [76], lentivirus [77], *Salmonella* [78], BCG [79], herpes simplex virus [80], Australian Flavivirus Kunjin [81], measles virus [82], mumps virus [83], rabies virus [84] and plant plastids [85]. Naked DNA was also proven to be an effective vaccine by Dr. Webster of St. Jude Children’s Research Hospital and Dr. Robinson of the University of Massachusetts when DNA-primed chickens and ferrets were protected from challenge with influenza virus, and has since been used as a vector in the HIV-1 vaccine field [86–89].

Some of the first recombinant vector systems were used to produce soluble viral antigens in culture*,* often formulated with adjuvants for pre-clinical and clinical research [90]. Vaccinations with soluble recombinant protein products typically elicit B-cell and CD4+ T-cell activities. With these products, CD8+ T-cell function is generally weak, because the classical mode of antigen processing for CD8+ T-cell responses requires endogenous protein expression [27]. Certain adjuvants have been shown to enhance CD8+ T-cell activity by promoting antigen cross-presentation, thus heightening the attraction of the soluble protein vaccine approach [91]. The DNA and live viral vector vaccines provide added benefit to soluble protein vaccines in that they instruct endogenous protein expression by the vaccinated host cell, and in the case of virus, can induce remarkably durable CD8+ T-cell, CD4+ T-cell and B-cell activities [27,92–94].

### 4.2. HIV-1 gene modifications

Molecular biology enables construction of multiple delivery vehicles and also assists the modification of vaccine antigens. Therefore, researchers have tested a great number of HIV or SIV antigen modifications. Genetic manipulations include the deletion of selected protein fragments [95], the creation of computer-designed sequences (e.g., ancestral, consensus, mosaic sequences [96–99]) and/or the alteration of post-translational modifications (e.g., removal of potential di-sulfide bonds or glycosylation sites [23,100]). In fact, proteins can be entirely scrambled to yield products with little similarity to their original template [101].

The study of modified proteins has been highly instructive, and has demonstrated that protein modifications may significantly impact antigenicity, both positively and negatively. Even when mutations are introduced into positions distant from a targeted epitope, they may alter epitope presentation. For example, the three dimensional structure upon which many B-cell epitopes are dependent, may be altered when a mutation is introduced at a distant site [102]. T-cell determinants can also be affected by mutations outside the target peptide, because the release of peptide for association with MHC depends on protein fragmentation, which in turn depends on disulfide bonds, protease cut-sites and glycosylation [103–105].

Debates continue as to how extensively epitopes should be modified from their natural context in candidate vaccines. It has been thought preferable to mimic the post-translational modifications typical of HIV-1-infected mammalian cells, discouraging the early use of recombinant baculovirus or yeast vectors [90,106]. Nevertheless, HIV-1 epitopes have since been expressed by numerous vectors that do not instruct post-translational modifications typical of the mammalian cell [70,71]. Discussions are ongoing as researchers strive to balance the flexibility of recombinant technology with their charge to mimic the natural epitopes of HIV-1.

## 5. Prime-boost vaccine regimens

### 5.1. Prime-boost with one or two delivery vehicles

The prime-boost strategy is routinely used in vaccination regimens to increase the magnitude of an immune response. Classical immune tests show that when the immune system is activated upon delivery of a foreign antigen, allowed to rest, and then reactivated, there can be an enormous improvement in both B-cell and T-cell responsiveness [27]. Boosting is relatively simple when a preformed antigen is used, but becomes more difficult when the same live virus vector is used repeatedly to direct antigen expression by host cells. In the latter instance, an anti-vector immune response (induced by the priming vaccine) can block efficacy of the boost. For example, when vaccinia virus is used to prime an immune response, vaccinia virus-specific immune responses can be so robust that a boost inoculation with vaccinia virus will not ‘take’. Essentially, the boost vaccine is quelled before it can instruct host cells to express foreign protein, and therefore provides little improvement to the vaccination regimen.

To overcome the vector-specific immunity induced by an initial prime, one can use a different vector for the boost. This strategy protects the boost vaccine from vector-specific antibodies and also protects infected host cells from vector-specific cytotoxic attack. One further advantage of the heterologous prime-boost strategy is that two different vectors may be selected to target two different lymphocyte subsets [107].

In the early 1990s, the value of the heterologous prime boost in the context of HIV-1 vaccinations was demonstrated by Hu *et al.* Briefly, investigators used a recombinant vaccinia virus that expressed an HIV-1 (BRU) envelope for the priming of small animals, and followed this with a purified HIV-1 envelope protein boost [108]. They next tested the heterologous prime-boost strategy in non-human primates. In this experiment, they demonstrated that an envelope vaccine administered by heterologous prime-boost was fully protective against SIV infection provided that the SIV antigens were identical between vaccine and challenge virus [109].

In the mid-1990s, our own group collaborated with researchers from the University of Massachusetts, to test a prime-boost regimen using DNA and vaccinia viruses each expressing HIV-1 envelope proteins. We demonstrated that although repeated inoculations with the recombinant DNA vaccine induced relatively weak responses, a single boost with recombinant vaccinia viruses improved immune responses by 1–2 orders of magnitude. This response also exceeded that which could be induced with vaccinia virus alone [110]. At the same time, Ramsay *et al*. tested DNA and fowlpox virus in a prime-boost regimen, with similar results [111–113].

The prime boost strategy has now been adopted by many research groups in HIV-1 and other fields [71,83,99,114,115]. There are many variations on the theme such as the use of a DNA prime and NYVAC boost [116], a DNA prime and adenovirus boost [117], a DNA prime and vesicular stomatitis virus boost [118], a mumps virus prime and a vesicular stomatitis virus boost [83], a DNA prime and protein boost [115,119], a DNA prime and recombinant phage boost [71], an MVA prime and a fowlpox boost [120] or an adenovirus prime and a protein, peptide or chimeric alphavirus replicon particle boost [121–124]. As new vectors are developed, these are often tested in prime-boost strategies. For example, adenoviruses from chimpanzees have been tested as a means to overcome the ad5-specific pre-existing immunity in humans that is known to hamper vaccine efficacy (as was the case in the disappointing Merck-sponsored STEP trial [7]). If used in a prime-boost protocol, the vector specific response induced by one serotype of adenovirus (e.g., adC6) need not inhibit the ‘take’ of a boost vaccine (e.g., adC7, [125,126]).

The prime-boost strategy with heterologous vectors is also showing promise in clinical trials, as indicated by the moderately successful RV 144 trial [3]. Another example of a prime-boost protocol in the clinic is the PAVE 100 study, redesigned as HVTN 505. This DNA-adenovirus prime-boost vaccine includes 3 HIV-1 envelopes (clades A, B, and C), as well as gag, pol and nef [127,128]. Still another prime-boost strategy uses DNA and MVA vectors. An early test of DNA-MVA was with recombinants expressing T-cell peptides. Results were discouraging in that immune responses were induced in only a small fraction of vaccinated individuals [4]. More recently, the DNA-MVA prime-boost protocol has been employed to deliver proteins rather than peptides [129–131], yielding better outcomes. A GeoVax DNA-MVA clade B vaccine is now in a Phase IIa trial, HVTN 205. The DNA component expresses gag, protease, reverse transcriptase, envelope, vpu, tat and rev sequences, while the MVA component expresses gag, protease, reverse transcriptase and envelope sequences [132]. Another group is using the DNA-MVA prime-boost strategy to present 4 envelopes plus rev, gag, pol and reverse transcriptase (described in more detail below [130,131,133–135]). An additional vaccine strategy forwarded by investigators at the University of Massachusetts Medical School and Advanced Bioscience Laboratories involves the use of a DNA-protein prime-boost regimen to deliver 5 envelope proteins from 4 different clades and gag [107,119,136,137].

As time progresses, researchers are becoming increasingly aware of the need to represent more than one virus isolate in HIV-1 vaccines [138,139], explaining the inclusion of 3, 4 or more envelopes in several current clinical vaccine formulations. The advancement of this strategy to clinical trials was prompted by a number of small and large animal pre-clinical studies comparing individual envelopes with envelope cocktails. Results demonstrated that the cocktails elicited qualitatively improved immune responses compared to single-envelope vaccines [130,133,134,140–143]. The strategy was also prompted by successes in other vaccine fields with the cocktail approach (e.g., influenza virus, rotavirus, papilloma virus, varicella virus and polio virus fields). Clinical trial data show that envelope cocktail vaccines can elicit responses against heterologous and diverse panels of HIV-1 [136,144,145]. While the heterologous target viruses are not precisely represented by sequences in the vaccines, there is sufficient similarity between at least one of the vaccine components and each target to render immune responses cross-reactive.

### 5.2. Three or more vectors in prime-boost protocols

In the late 1990s, we first tested a prime-boost-boost strategy, using three different vector systems to express HIV-1 envelope proteins [145–149]. Our vectors included recombinant DNA (D), recombinant virus (V, in this case vaccinia virus) and recombinant protein (P). Results demonstrated that three vaccinations with different vectors elicited durable B-cell and T-cell responses [147,148]. Research with three-vector systems have since been conducted by other groups in the context of HIV-1 and non-HIV-1 vaccines [150].

**Figure 1.**
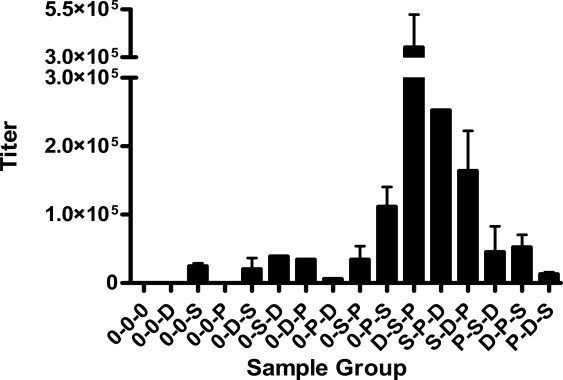
Durable antibody responses elicited by successive HIV-1 envelope immunizations with three different recombinant delivery systems. Female C57BL/6 mice were purchased from the Jackson Laboratory (Bar Harbor, ME) and housed under specific pathogen-free conditions in a BL1 or BL2+ containment area at St. Jude Children’s Research Hospital animal facilities, with adherence to AAALAC Guidelines. Mice were grouped (n = 3) for immunizations with UG92005 (UG) envelope using recombinant vectors: (i) DNA (‘D’, 100 μg, carrying a gp140 envelope sequence) administered i.m. in the gastrocnemius muscles, (ii) CHO-derived recombinant UG envelope protein (‘P’, 2 μg, 100 μl gp140 envelope in PBS mixed with 100 μl CFA) administered i.p., and (iii) SeV (‘S’, 10^4^ plaque forming units, carrying a gp120 envelope sequence for expression in infected cells) administered i.n.. Groups of mice received one, two or three of the recombinant vectors (or no vaccine, ‘0’), administered with one month intervals. Eight months after the completion of inoculations, ELISAs were performed with serially diluted serum samples. Antibody binding titers were determined using Prism Software (Non-Linear Regression, GraphPad Prism®, San Diego, CA). Mean titers and standard errors are shown. Some data points represent fewer than 3 animals due to mouse death or non-conformance of data to the non-linear regression curve (O-S-D, O-D-P, O-P-D, O-S-P, S-P-D, P-S-D). To perform the ELISA, 96-well plates (BD Biosciences, Franklin Lakes, NJ) were coated overnight at 4 °C with 2 μg/ml of purified CHO-derived UG gp140 envelope protein in PBS. The plates were washed three times with 0.05% Tween 20 in PBS, blocked with 1% BSA/PBS at room temperature for 1 h, and washed an additional three times. Samples were serially diluted in PBS to a final volume of 50 μl and were incubated in wells for 2 h at room temperature. After three washes, alkaline phosphatase-conjugated anti-mouse IgG1 (50 μl/well; Southern Biotechnology Associates, Birmingham, AL) diluted 1/1000 in 1% BSA/0.05% Tween 20/PBS was added for 1 h at room temperature. Following five washes, the assay was developed with 75 μl/well of p-nitrophenyl phosphate (Sigma-Aldrich) substrate (1 mg/ml in diethanolamine buffer) and was read at OD_450nm_ after 1 hour at 37 °C.

What is the best order of vaccinations when three vectors are used? When using recombinant vaccinia virus as the recombinant virus, we found that the order D-V-P was best [146]. We then asked if the optimal order of vector delivery would change if Sendai virus (SeV) was used as the viral component. To answer this question, we grouped mice to receive one, two or three of the D, SeV and P vaccines in different orders. Each vector was a UG92005 envelope recombinant. After vaccinations, mice were rested for eight months and then anti-envelope antibodies were tested in a UG92005-based envelope ELISA. The results of ELISAs are shown in Figure 1.

We noted that virtually all of the animals that received recombinant SeV generated long-term immunity, a result which highlighted the well known association of live virus vaccines with durable immune responses [92–94,151]. The preliminary study results in Figure 1 further showed that the more durable antibody immune responses were elicited when the SeV immunization was administered as the first or second inoculation (see D-S-P or S-P-D) rather than as the third immunication (see D-P-S or P-D-S). The result was confirmed in a repeat experiment. This phenomenon might be explained by our previous finding that a robust pre-existing T-cell response toward HIV-1 envelope can significantly inhibit the growth or ‘take’ of recombinant SeV [152]. The situation of vaccine inhibition is similar to that described previously in that a vector-specific response can quell the ‘take’ of a live virus boost. The difference in this case is that the pre-existing immune response targets the HIV-1 envelope passenger gene product rather than the delivery vehicle. If the immune response to the passenger gene product is too strong (in this case following D or P vaccines), the SeV vaccine may not ‘take’, yielding an inferior overall outcome. Results highlight the complexities of the prime-boost strategy and demonstrate how each inoculation can impact the efficacy of the next.

Yu *et al*. have also examined a three-vectored prime-boost-boost regimen, in this case using DNA, adenovirus and SeV recombinants expressing gag antigens. They found that the vaccines delivered in the order DNA-SeV-adenovirus yielded slightly higher gag-specific CD8+ T-cell activities than vaccines delivered in the order DNA-adenovirus-SeV [150,153].

### 5.3. Complex factors impact prime-boost vaccine outcomes

A great number of factors will impact the final outcome of a prime-boost strategy, due to the important interplay between responses induced by each inoculation. A subtle balance must therefore be struck. On the one hand, the activation of naïve cells by a priming vaccine is desired. On the other hand, an overly robust primary immune response may be counter-productive if it inhibits the efficacy of a boost. DNA and virus vaccines are perhaps most sensitive to pre-existing immune responses, because they must transfect or infect host cells prior to production of the desired antigen. If/when host cells are susceptible to immune attack due to pre-existing immunity, the boost function may be reduced or eliminated. It is perhaps the case that a protein boost is most resilient to the effects of pre-existing immunity, because the antigen is preformed and its expression is not dependent on infection or transfection of the host cell.

Not only will the immune response toward prime-boost vaccination depend on the selection of recombinant vectors and the order in which they are administered, but vaccine dose will also affect outcome. Again, a balance must be reached to successfully ‘prime’ the immune response without inhibiting the boost. The selection of a priming dose can sometimes be counter-intuitive in that an increase in dose does not always improve immunogenicity. This is because high-dose priming antigens can in some cases hinder a response to the boost inoculation and can also hinder the establishment of long-term immune memory [154,155]. In a hepatitis vaccine clinical study, researchers found that a dose as small as 2 μg DNA could be effective as part of a prime-boost regimen [156].

To complicate vaccine formulation further, it is noteworthy that a dose study in one species need not predict vaccine outcome in a second. As an example, a DNA dose escalation study in mice suggested that increases in DNA dose would improve immune activities, yet in macaques a 10-fold DNA dose escalation was not advantageous [157,158]. The situation becomes more complicated with each experimental change. Another example is provided by the opposing outcomes in studies of DNA-adenovirus prime-boost regimens in macaques. Whereas research in one system demonstrated complementary effects of a DNA prime and adenovirus boost [117], a more recent protection study showed no advantage conferred by a 400 mg DNA prime administered prior to an adenovirus boost [159].

Among the many factors influencing outcome in a prime-boost study are: (i) the vaccine vectors, (ii) vaccine doses, (iii) the interval between prime and boost, (iv) virus tropism for the host species, (v) non-vaccine sources of pre-existing immunity, (vi) the type of desired immune activity (e.g., B-cell *versus* T-cell, Th1 *versus* Th2), and (vii) the timing of desired immune function (acute *versus* long-term). When prime-boost regimens are newly designed, each factor deserves attention to ensure vaccine efficacy.

## 6. Changing antigens during a prime-boost regimen

### 6.1. Introducing variant antigens in prime-boost vaccine protocols: lessons from the influenza virus vaccine field and original antigenic sin

The influenza virus, like HIV-1, encompasses a large diversity of viral proteins. The review of nucleic acid or amino acid sequences from influenza virus variants may be mind-boggling to the vaccine developer, but researchers have grouped viruses by antigenicity rather than by sequence to produce effective vaccines.

Influenza virus vaccine developers must accommodate antigenic drift (moderate changes in viral proteins) as well as antigenic shift (major changes in viral proteins incurred when the virus jumps species) [160]. To deal with these temporal changes, vaccine developers produce new vaccines annually. They also produce novel vaccines in response to shifting viral variants, as for the recent H1N1 swine flu pandemic [161]. Humans who receive vaccines each year are exposed to ‘new’ heterologous influenza virus hemagglutinin and neuraminidase proteins with each boost inoculation.

Original antigenic sin (OAS) was demonstrated decades ago in the context of immune responses toward influenza antigens. When humans or experimental animals experienced infections with related influenza viruses, their sera repeatedly showed higher neutralizing titers against the earlier encountered (‘original’) virus strain [162,163]. The common theme of pre-existing immunity may be central to the explanation of OAS. In this case, the immune system has memory for a portion of (but not all) epitopes on the challenge virus and may therefore hamper boost function. The concept is similar to that described above in the context of vector-specific or HIV-1-envelope-specific activities which can clear and weaken the boost inoculum. As before, clearance can be mediated by both humoral (B-cell) and cellular (T-cell) responses. In the case of humoral immunity, pre-existing antibodies may recognize and clear virus particles. In the case of cellular immunity, effectors can kill virus-infected targets that display viral peptides on membrane surfaces in the context of MHC. Together, immune effectors can reduce antigen load and durability of the boost. In this instance, whereas the transient appearance of boost antigen may suffice to drive re-activation and replication of memory cells responsive to the ‘old’ epitopes, the antigen load and persistence may be insufficient to activate naïve lymphocytes responsive to ‘new’ epitopes. The outcome is an improved response toward ‘old’ or ‘original’ epitopes, but a relatively weak response toward epitopes that are newly presented.

Is OAS absolute? In a recent influenza virus study in mice, researchers confirmed the phenomenon of profound OAS [164]. In contrast, a recent study in humans revealed the opposite result [165]. In the latter case, humans who were vaccinated with the seasonal influenza virus vaccine responded better to ‘new’ *versus* ‘old’ epitopes. Results supported the theory upon which influenza virus vaccines are based, that the quality of immune responses can be improved by successive immunizations with related, but dissimilar influenza viral antigens. Contrasting results in OAS studies again illustrate that the rules driving immune responses to a prime-boost vaccine are not simple. As stated above, outcomes will depend on complex variables including (i) vaccine dose, (ii) interval between first and second antigen exposure, and (iii) relatedness of epitopes between old and new antigens.

### 6.2. T-cells responsive to an epitope shared by prime and boost vaccines can assist B-cells responsive to a unique epitope in the boost

Even though a vigorous pre-existing immune response may limit the load and persistence of boost antigens, memory cells can also play a positive role. Specifically, the CD4+ T cells that are primed by a first dose of vaccine may ‘help’ naïve lymphocytes to respond to novel epitopes in the boost inoculum.

**Figure 2.**
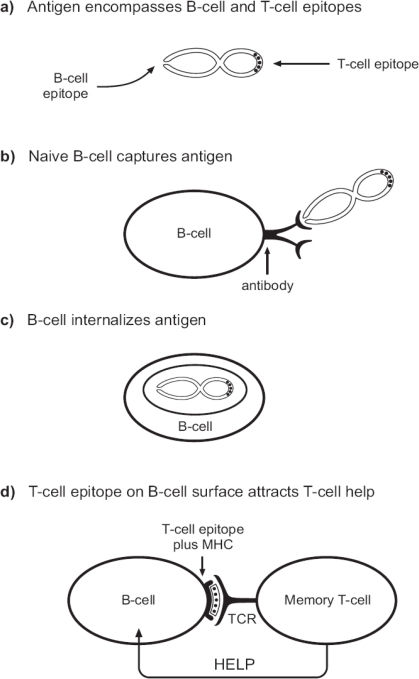
Cognate B-cell:T-cell interactions support T-cell help. This cartoon illustrates a situation in which a B cell and T cell recognize different determinants on the same particle (panel A). In this circumstance, the B-cell antibody may capture antigen via a lock-and-key type interaction (panel B). The antigen is then fragmented (panel C), after which the T-cell epitope is presented on the B-cell membrane as a complex with MHC. The peptide-MHC complex is bound by the T-cell receptor, after which ‘help’ may be relayed from the T cell to its B cell partner (panel D).

T-cell help is conferred by a variety of mechanisms including secretion of interleukins and activation of antigen presenting cells [27]. In Figure 2 is illustrated the cognate B-cell:T-cell interactions that can enhance naive B-cell stimulation. Briefly, when a naïve B cell encounters antigen for the first time (Figure 2A), its antibody will bind and internalize the antigenic protein or particle (Figure 2A–C). Antigens are then fragmented for ultimate display on the cell surface in association with MHC class II. The peptide-MHC complex can then be bound by the TCR of a memory T cell (Figure 2D). Cognate B-cell:T-cell interactions then trigger the relay of ‘help’ (e.g., interleukin 4) for support of B-cell activation. It is noteworthy that the epitopes recognized by the B cells and T cells need not be the same. The T-cell epitope may be shared by the prime and boost vaccines, whereas the B-cell eptiope may be unique to the boost. It is only necessary that the two epitopes are linked on the antigen or particle to which antibody is bound [27]. The phenomenon can be observed when mice are primed with a vaccine *in vivo*, after which lymphocytes are exposed to a new determinant(s) linked to that vaccine *in vitro*. In this scenario, naïve B cells with specificity for the new determinant(s) are activated when they receive ‘help’ from the vaccine-induced memory T cells [166,167].

The lesson from the above experiments is that a pre-existing immune response elicited by a priming vaccine can have both positive and negative effects on naïve responses to novel epitopes in a boost. The balance yields striking OAS in some circumstances, and relatively little OAS in others.

### 6.3. HIV and OAS

In 1991, Klinman *et al*. [168] described a study in which mice received sequential inoculations with gp120 proteins from independent isolates of HIV-1. The specificities of vaccine-induced plasma cells were then tested by positioning cells between two antigen coated surfaces. Secreted antibodies were examined by spot analyses for simultaneous recognition of two different antigens. Researchers found that OAS was a factor, just as had been the case in the influenza virus studies, but was not absolute; immune responses toward shared determinants were dominant, but responses to new antigens, presented in the boost inocula, were also observed.

Zhan *et al*. later evaluated T-cell activities toward a heterologous prime-boost regimen in mice after successive immunizations with two different HIV-1 envelope gp140 constructs [169]. In this case, the two gp140 antigens 1007 and UG92005 carried very different immunodominant peptides [170,171]. Despite the inoculation of mice with one immunogen (1007) before the other (UG92005), T-cell responses toward the unique epitopes on each of the antigens were generated [169].

Wang *et al*. [172] also tested a heterologous prime-boost approach, in which peptides were used as a prime and a recombinant vaccinia virus was used to deliver the boost. They found that the first immunization had insignificant effect on the CD8+ T-cell dominance hierarchies induced by the second. Taken together, these results suggest that novel proteins and epitopes can be introduced in the boost phase of a prime-boost regimen to improve the quality of the HIV-1-specific response in both B-cell and T-cell populations.

As stated above, the vaccine evaluated in the recent RV 144 clinical trial in Thailand presented heterologous proteins upon prime and boost. This prime-boost vaccine demonstrated a hint of protection whereas individual vaccines were not protective when administered alone [173]. While the precise mechanism of protection is not yet known, it is likely that the combination of ‘old’ and ‘new’ responses toward antigens in prime and boost vaccines lent to the moderate success.

Perhaps the best example of incomplete OAS is demonstrated by infections with SIV, SHIV or HIV. As described above, an infected subject is exposed to a variety of viral variants over the course of time and responds with an ever-increasing quality of immune activity. That immune activity is often sufficient to confer protection against virus from an exogenous source [14,41,47,174,175]. It is worth contemplating that if a safe vaccine could recapitulate the immune responses induced by the successive antigens presented by natural infection, uninfected individuals might be fully protected from HIV-1.

## 7. Changing both vectors and antigens during heterologous prime-boost immunizations in HIV-1 vaccine clinical trials

Several clinical studies have now tested strategies that change both vectors and antigens during prime-boost regimens, as was the case in the RV 144 trial. For example, Gudmundsdotter *et al*. are currently testing a DNA vaccine that expresses envelopes from Subtypes A, B and C (A92UG031, LAI and 92BR025), rev from subtype B, gag from subtypes A and B, reverse transcriptase from subtype B, and a boost vaccine that is an MVA-Chiang Mai double recombinant (MVA-CMDR)(HIV MVA) expressing envelope and gag/pol sequences from Thai isolates CM235 and CM240 [131].

Our own research has involved clinical testing of a multi-envelope heterologous prime-boost approach using DNA, virus (vaccinia virus) and purified protein recombinants. The selection of envelopes for inclusion in our vaccine was based on (i) differing patterns in antibody binding studies [176], (ii) longitudinal capture from infected persons [138], and (iii) differing clades. This strategy was first tested in macaques and found to confer protection against a heterologous SHIV challenge [177] which was not represented by sequence in the vaccine. To test the concept in humans, we first demonstrated the safety of each of the three vectors D, V, and P in FDA- and IRB-approved phase I clinical studies [144,145,178]. We next designed a protocol, approved by the FDA and our local IRB, to vaccinate individuals with dozens of envelopes. Six successive immunizations (D-D-V-P-D-P) separated by 1 month intervals, were planned for each individual. The D vaccine included 51 recombinant plasmids each expressing a different envelope protein, administered as an i.m. inoculation of 100 micrograms; the vaccinia virus vaccine included 23 recombinant viruses, each expressing a different envelope protein (a subset of which were shared with the D vaccine), administered by s.q. inoculation with 10^7^ total plaque forming units; the recombinant CHO-derived protein vaccine was 100 microgram purified envelope from HIV-1 isolate UG92005 (shared with D and V vaccines), administered i.m. as a formulation with 500 micrograms alum. The single protein immunization was administered to enhance both B-cell and T-cell responses. We emphasize that based on the mechanism described in Figure 2, a T cell which has responded to one envelope immunogen can ‘help’ a B cell which has responded to another, provided that the B cell displays the T-cell epitope on its surface.

Based on a local administrative decision, only three individuals were ultimately enrolled in the study. The first participant received all 6 inoculations (D-D-V-P-D-P); the second participant received four inoculations (D-D-V-P), and the third recipient received three inoculations (D-D-V). All inoculations were well tolerated. Adverse events that were possibly, probably or definitely vaccine related were grade 1 only.

**Figure 3.**
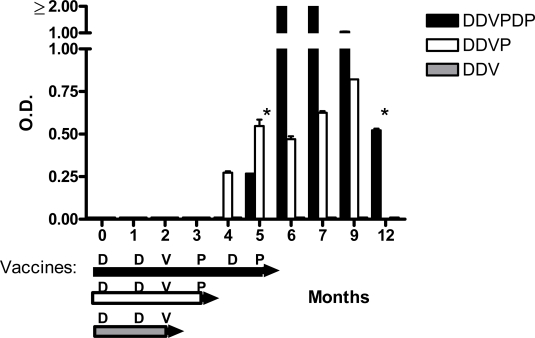
Antibody responses elicited by heterologous prime-boost vaccination of humans. Three study participants received three different vaccination regimens. Study Participant 1 (black bars) received D (month 0), D (month 1), V (month 2), P (month 3), D (month 4) and P (month 5). Participant 2 (clear bars) received the first four inoculations and Participant 3 (grey bars) received the first three inoculations. Sera were collected and tests were conducted with the Abbott enzyme-linked immunosorbent assay (HIVAB HIV-1/HIV-2 (recombinant DNA) Abbott Laboratories, Abbott Park, IL, USA). Asterisks indicate an absence of sample.

Preliminary tests of envelope-specific immune responses were conducted with the Abbott enzyme-linked immunosorbent assay (HIVAB HIV-1/HIV-2 (recombinant DNA) Abbott Laboratories, Abbott Park, IL, USA). This standardized assay is used worldwide as a diagnostic for HIV-1 infection and was conducted in the Clinical Pathology Department of St. Jude Children’s Research Hospital. There is a cut-off score for each test (ranging between 0.1 and 0.2 OD_450nm_). Absolute responses for HIV-1-infected persons in this test can be variable and often reach the assay peak value of ≥2. In Figure 3, results are shown for the three vaccinees.

Participant 1 who received all six inoculations, generated the best response. An Abbott-positive response was first identified in this volunteer one month after the fifth immunization (D-D-V-P-D). One month after the sixth immunization (P), the participant exhibited peak absorbance levels in the Abbott assay (OD_450nm_ ≥ 2). Significant responses persisted for at least seven months following the last inoculation.

Participant 2, who received four immunizations (D-D-V-P) also generated significant envelope-specific activity. This response continued to improve throughout the observation period, but did not achieve peak values (within the observation period which ended 6 months after the final immunization).

Participant 3, who received only D-D-V did not generate a positive response in the Abbott assay. Apparently, the combination of at least 4 immunizations and 3 vector systems was advantageous, distinguishing the positive responses in Participants 1 and 2 from the negative response in Participant 3.

The antibodies with the strongest immune activity (from Participant 1 taken 1 month following the completion of all vaccinations) were also tested for neutralizing activity in a preliminary GHOST cell-based assay [145]. To conduct this study, antibodies were first purified over a protein G column to remove non-specific factors. Although immunoglobulin purification is not standard practice in the field, it may be essential to avoid the non-specific inhibitory or enhancing effects of serum components in the HIV-1 neutralization assay [179–182]. Such factors are particularly problematic when sera are tested at high concentrations and/or when results from parallel analyses with panels of unselected negative control sera are not available. In our study, of eight tested heterologous viruses, four viruses were neutralized at the 50% level by samples taken post-vaccination (diluted 1:5 relative to the original serum value), but not pre-vaccination. Positive tests were with viruses HIV-1_IIIB_ (CXCR4), HIV-1_30e_ (CXCR4), HIV-1_SF2_ (CCR5) and HIV-1_92HT593_ (CCR5), but not viruses HIV-1_96ZM651_ (CCR5), HIV-1_ZM53M_ (CCR5), HIV-1_92UG029_ (CCR5) and HIV-1_93UG082_ (CCR5). The neutralization of four heterologous viruses was promising and indicated the sharing of antigenic structures between at least one envelope in the vaccine and envelopes on test viruses.

The relevance of neutralizing responses to protection *in vivo* is not known. Currently, there are no clear correlates of protection in the HIV-1 field. In fact, antibodies can demonstrate a much improved protective capacity *in vivo versus in vitro*. For example, in a macaque study, passive transfer of hyperimmune sera to naïve animals was fully protective against SIV, even when the same antibodies showed no neutralizing activity against the same challenge virus *in vitro* [183]. The neutralization assay is clearly able to measure some vaccine-induced antibody potentials, but misses critical antibody functions such as antibody-dependent cell-mediated cytotoxicity (ADCC) and antibody-dependent cell-mediated virus inhibition (ADCVI, [184–186]). Taken together, the preliminary clinical results with D-V-P vaccines demonstrate a degree of immune breadth and encourage continued testing of the vaccine concept with larger participant groups and larger virus panels. Preliminary results also reveal safety and immunogenicity of the vaccine in humans and show that responses are durable.

What might be the mechanism responsible for antibody (and T-cell) durability, particularly in the context of a live virus infection (a single vaccinia virus inoculation elicits antibody responses that can persist for more than 70 years in humans [92–94,187–190])? Immune response durability has long been a topic of much debate. Some researchers argue that antigens (particularly viral antigens) are maintained *in vivo* for the long term and constitutively drive B cells to end-differentiate into antibody forming cells [191]. Other researchers, however, have shown that when B cells have lost their capacity to recognize an antigen, they still persist. The latter feature was best illustrated using a cre/lox recombination system with which antibody genes were intentionally altered among memory B cells *in vivo*. B-cells that had lost their capacity to recognize the priming antigen were nonetheless maintained [192]. These results suggest that immune responses are maintained by a combination of factors, including antigen, memory B cell, and antibody-forming cell persistence [191].

The proof-of-concept findings described by ourselves and others in pre-clinical and phase I/II clinical trials cannot predict protective efficacy, but in combination with the recent hint of success in the RV 144 trial, encourage further advancement of vaccine strategies that combine heterologous vectors and heterologous antigens in a prime-boost approach.

## 8. Conclusion

The heterologous prime-boost vaccination strategy can be used to harness robust and durable immune activity, and is particularly useful as a means to circumvent vector-specific inhibitory effects. In the context of a heterologous prime-boost, there are also inhibitory effects directed toward the passenger gene which can lend to ‘original antigenic sin’. Fortunately, this phenomenon is not absolute. The final outcome of prime-boost vaccinations is complex and will be influenced by multiple factors including: (i) the vaccine vectors, (ii) the vaccine doses, (iii) the interval between prime and boost, (iv) virus tropism for the host species, (v) non-vaccine sources of pre-existing immunity, (vi) the type of desired immune activity, and (vii) the timing of desired immune function. Despite the complexities posed by heterologous prime-boost vaccine protocols, the strategy holds enormous promise and might ultimately prevent the morbidity and mortality caused by HIV-1.
